# The impact of the COVID-19 pandemic on colorectal and gastric cancer diagnosis, disease stage and mortality

**DOI:** 10.3389/fmed.2022.954878

**Published:** 2022-09-26

**Authors:** Naim Abu-Freha, Reut Hizkiya, Muhammad Abu-Abed, Tal Michael, Binil Mathew Jacob, Keren Rouvinov, Doron Schwartz, Avraham Reshef, Uri Netz, Ilia Pinsk, Ohad Etzion

**Affiliations:** ^1^The Institute of Gastroenterology and Liver Diseases, Soroka University Medical Center, Beer Sheva, Israel; ^2^Faculty of Health Sciences, Ben-Gurion University of the Negev, Beer Sheva, Israel; ^3^Internal Medicine Division, Soroka University Medical Center, Beer Sheva, Israel; ^4^Department of Public Health, The Faculty of Health Sciences, Ben-Gurion University of the Negev, Beer Sheva, Israel; ^5^Medical School for International Health, Ben-Gurion University of the Negev, Beer Sheva, Israel; ^6^The Legacy Heritage Oncology Center and Dr. Larry Norton Institute, Soroka Medical Center, Beer Sheva, Israel; ^7^Department of Surgery, Soroka University Medical Center, Beer Sheva, Israel

**Keywords:** COVID-19, colon cancer, gastric cancer, disease stage, cancer

## Abstract

**Background:**

Since the outbreak of COVID-19, a significant decline in endoscopic procedures has been observed.

**Aims:**

We investigated the change of incidence, clinical characteristics, disease stage and mortality of patients with gastric cancer (GC) or colorectal cancer (CRC) diagnosed in 2020 compared to the pre-pandemic year 2019.

**Methods:**

Demographic, clinical and laboratory data on all patients diagnosed with GC or CRC at the Soroka University Medical Center were retrospectively collected and compared. Number of cases, time of diagnosis, clinical presentation, staging at diagnosis and mortality rates were compared.

**Results:**

Two hundred sixteen patients were diagnosed with CRC in 2019, whereas only 162 were diagnosed in 2020 (25% reduction), while 36 GC diagnoses were made in 2019 compared to 24 in 2020 (33% reduction). The age-adjusted incidence was calculated to be 24.28 for CRC and 5.0 for GC in 2020 compared to 29.93 and 5.32 in 2019, respectively. CRC patients had a significantly lower rate of rectal bleeding as their presenting symptom in 2020 compared with 2019, 8.1 vs. 19% (*p* = 0.003), but higher rate of diarrhea as their presenting symptom, 4.3 vs. 1% (*p* = 0.044). No significant differences regarding other presenting symptoms, comorbidities, surgery or mortality rates were found between the groups diagnosed in 2019 or 2020.

**Conclusion:**

A decrease in GC and CRC incidence was observed during the year 2020; lower rate of rectal bleeding and higher rate of diarrhea as presenting symptoms were noted in 2020, but no significant difference was found regarding other presenting symptoms, disease stage, surgery or mortality.

## Introduction

The ongoing novel corona virus (COVID-19) pandemic originated in Wuhan, China in December 2019. Over the following months, the virus spread throughout the world leading to changes regarding the approach to endoscopic procedures in the year 2020 (1, 2). This change in the roadmap of screening and diagnostic endoscopic procedures during the COVID-19 pandemic has been influenced by a variety of stakeholders including: national health authorities, international endoscopy associations, physician and patient preferences and others. The lack of knowledge and uncertainty in this jarring new reality were further compounded by the necessity to maintain safety of both the patients and professionals. The recommendations for endoscopic procedure timing and prioritization were altered continuously in response to the dynamic changes in disease spread and viral infectivity throughout the globe. This led to widespread cancellations or postponements of procedures and a global reduction in elective endoscopic procedures as well as non-essential gastrointestinal office activity. Several guidelines and updates were published by endoscopic associations to stratify the clinical indications or organizational prioritization for gastrointestinal procedures, including endoscopy, on the basis of infection prevention and control and ensuring the safety of the health care professionals and patients (1, 2).

One of the most highly impacted decisions by national authorities and health policy makers were implementation of lockdowns that restricted mobility within and between cities for residents of the country. For example, in Israel during 2020, there were three nationwide lockdowns (March/April, September/October and December/January 2021). The first lockdown was the tightest with very limited mobility and as such restricted endoscopic procedures performed in the hospital, permitting only emergency procedures, and postponing or canceling all elective endoscopic diagnostic procedures.

During 2020, a significant decrease in endoscopic procedures was reported in many countries. Lahat et al. all showed a reduction of 39% of esophagogastroduodenoscopy (EGD) and 57% of colonoscopy procedures, while another study of 15 collective data from Dutch hospitals reported a reduction of 57% of EGD and 45% in colonoscopy procedures performed during this period (3–5). Current knowledge is limited regarding the effect of this decrease on cancer diagnosis, disease stage at diagnosis and mortality.

The objective of the present study is to investigate the effect on incidence of diagnosis and clinical characteristics of colorectal cancer (CRC) and gastric cancer (GC) of the pandemic year 2020 in comparison to the previous unconstrained 2019.

## Methods

This is a retrospective study comparing patients diagnosed with CRC and GC in the years 2019 and 2020 at Soroka University Medical Center (SUMC), Beer Sheva, Israel. The study was carried out in accordance with the principles of the Helsinki Declaration. The study protocol was approved by the SUMC IRB Committee, approval number 558-2020-SOR.

### Study population

A search for the diagnosis of CRC and GC diagnosis was performed on three different databases: the cancer registry of the SUMC cancer center, endoscopy reports of the gastroenterology department, and a hospitalization diagnosis of CRC or GC in the Internal Medicine Division or surgical departments at SUMC. We included all patients with a new confirmed diagnosis of CRC or GC according to tissue diagnosis during the years 2019 and 2020. The SUMC is a tertiary center of 1,100 beds and provides health care in the southern district in Israel, the data of the same population were compared during the study period.

### Data collection

Demographic data collected from patient medical records included age, age at diagnosis, gender, date of diagnosis and ethnicity; Clinical data collected included symptoms at presentation, comorbidities, Charlson comorbidities score, localization of the cancer (right colon, transverse colon, left colon, and rectum for CRC, and antrum, body, and cardia for gastric cancer), stage at diagnosis and mortality. The data of patients with CRC or GC diagnosed in the year 2019 were compared with those diagnosed in 2020.

### Statistical analysis

Patient characteristics were presented as mean ±SD for continuous variables and as percentages for categorical variables. Categorical variables were compared using the chi-square test. Numeric variables were examined with the student *t*-test as well as Kruskal-Wallis for normally distributed continuous and non-normally distributed variables respectively. Incidence rates were adjusted for age group, and calculated as the yearly number of new cases divided by the mid-year average size of the age group population living in the southern district of Israel. Population data was retrieved by the Israeli Central Bureau of Statistics online database (www.cbs.gov.il). All-cause mortality data were collected until Novermver 31^st^, 2021 resulting in a follow-up period of 11–23 months. *P* < 0.05 were considered statistically significant. All statistical analysis was performed using “R statistics” (R Core Team, 2019) using the packages “lubridate,” “dyplr,” and “ggplot2.”

## Results

### Colon cancer

**Figure 1:**
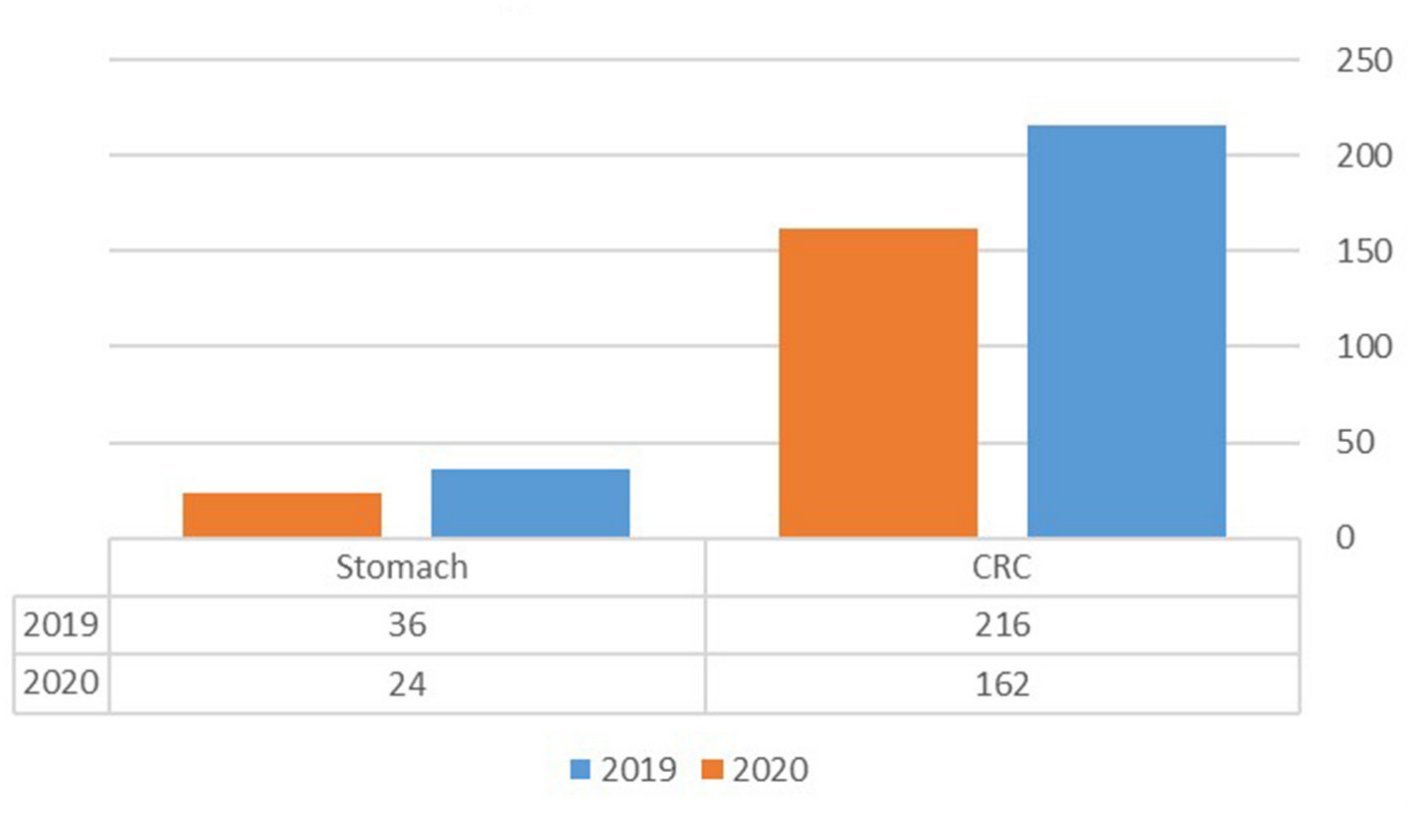
Number of patients diagnosed with CRC or gastric cancers during the years 2019 and 2020.

A total of 378 patients with CRC were detected during the study period, of whom 216 (57.1%) were diagnosed in 2019 and 162 (42.9%) in 2020 (Figure 1). The age-adjusted incidence of CRC was calculated as 29.9 cases per 100,000 residents per year in 2019 compared to 24.28 cases in 2020 (*p* = 0.377). The clinical characteristics, comorbidities and staging of the included patients are summarized in Table 1. Of all CRC patients, 59 (27%) were diagnosed with rectal cancer in 2019 compared with 30 (19%) patients in 2020. CRC patients had significantly lower rate of rectal bleeding as presenting symptom in 2020 compared with 2019, 8.1 vs. 19% (*p* = 0.003), but higher rate of diarrhea as presenting symptom, 4.3 vs. 1% (*p* = 0.044). No significant difference regarding other presenting symptoms such as abdominal pain, weight loss, fecal occult stool testing, change of bowel habits, or constipation was found between the groups. No statistically significant differences were found in comorbidities, Charlson comorbidities score, surgical treatment or mortality rate for patients diagnosed in 2019 vs. 2020. Two cases of COVID-19-related mortality were reported in 2020.

**Table 1A:** Demographic and clinical characteristics of patients with CRC in the years 2019 and 2020.

**Characteristics**	**Diagnosed 2019** ***n* = 216**	**Diagnosed 2020** ***n* = 162**	***P*-value**
Age (mean±SD)	71.16 ± 12	69.27 ± 12.85	0.15
Age at diagnosis (mean±SD)	69.41 ± 12.20	68.10 ± 12.79	0.33
Gender-male	113 (52)	89 (55)	0.6
Ethnicity - Bedouin	14 (6.5)	10 (6.2)	>0.9
**Symptoms**			
Abdominal pain Rectal bleeding Weight loss Fecal occult stool testing Change of bowel habits Constipation Diarrhea Polyp follow up Family history of CRC Anemia	53 (25) 40 (19) 14 (6.7) 34 (16) 9 (4.3) 15 (7.1) 2 (1) 5 (2.4) 0 30 (14)	55 (34) 13 (8.1) 4 (2.5) 23 (14) 3 (1.9) 8 (5) 7 (4.3) 5 (3.1) 3 (1.9) 28 (17)	0.061 0.003 0.063 0.6 0.2 0.4 0.044 0.9 0.081 0.4
Tumor site Colon Rectum	157 (73) 59 (27)	132 (81) 30 (19)	0.05
**Localization**			0.060
Right colon Left colon Rectum	75 (34.7) 82 (38) 59 (27.3)	73 (45.1) 59 (36.4) 30 (19.6)	
**Staging**			
I II III IV X	34 (16) 67 (31) 56 (26) 49 (23) 5 (2.3)	23 (14) 44 (27) 42 (26) 44 (27) 3 (1.9)	0.7 0.4 >0.9 0.3 < 0.9
Death	67 (31)	39 (24)	0.2
Age at death (mean ± SD)	75.71 ± 11.94	74.37 ± 12.13	0.58
Charlson score (mean ± SD)	11.89 ± 3.24	12.08 ± 2.61	0.79
**Comorbidities**			
CIHD CHF CVA PVD DM COPD Liver disease CRF Dementia HTN Dyslipidemia Smoking Obesity Family history of CRC	34 (16) 18 (8.4) 22 (10) 12 (5.6) 80 (37) 10 (4.6) 9 (4.2) 18 (8.3) 8 (3.7) 111 (51) 81 (38) 39 (18) 23 (13) 24 (15)	22 (14) 8 (4.9) 8 (4.9) 6 (3.7) 46 (29) 4 (2.5) 3 (1.9) 9 (5.6) 7 (4.3) 81 (51) 59 (36) 32 (20) 17 (10) 14 (11)	0.6 0.2 0.062 0.4 0.079 0.3 0.2 0.3 0.8 >0.9 0.8 0.7 0.4 0.3

CIHD, Chronic Ischemic Heart Disease; CHF, Congestive heart disease; CVA, cerebrovascular disease; PVD, peripheral vascular disease; DM, diabetes mellitus; COPD, chronic obstructive pulmonary disease; CRF, chronic renal failure; HTN, hypertension.

### Staging of CRC

34 patients (16%) were diagnosed at stage I in 2019 compared to 23 (14%) in 2020, while 49 (23%) were diagnosed at stage IV in 2019 compared with 44 (27%) in 2020. No statistically significant differences were found regarding the disease stages at diagnosis (Table 1). The subgroups of the disease stage were also compared and no significant differences were found.

### Timing and treatment of colorectal patients

The timing and type of treatments for patients with colorectal cancer are summarized in Table 1B.

**Table 1B:** Timing and treatment of colorectal cancer patients.

**Characteristics**	**Diagnosed 2019**	**Diagnosed 2020**	***P*-value**
	***n* = 216**	***n* = 162**	
Time from diagnosis to surgery mean±SD, days	*n* = 162	*n* = 125	
	32.84 ± 37	27.01 ± 38.4	0.194
Time from diagnosis to surgery after neoadjuvant radiotherapy mean±SD, days	*n* = 16	*n* = 6	
	148.94 ± 100	112.38 ± 73	0.434
Time from surgery to oncologic consultation mean±SD, days	*n* = 128	*n* = 121	
	35.16 ± 20.16	37.81 ± 21.4	0.316
Time from surgery to chemotherapy begin	*n* = 48	*n* = 54	
	63.15 ± 31.3	70.50 ± 55	0.419
Treatment surgery	167 (78)	125 (79)	>0.9
Palliative treatment	17 (7.9)	10 (6.3)	>0.9
Any chemotherapy	90 (41.7)	78 (48.1)	0.209
Any biological therapy	16 (7.4)	23 (14.2)	0.032
Neoadjuvant radiotherapy	19 (8.8)	8 (4.9)	0.149
Treatment protocol			
Capecitabine plus oxaliplatin (Xelox protocol)	28 (13)	34 (21)	0.037
Folinic acid, fluorouracil and oxaliplatin (Folfox protocol)	34 (15.7)	24 (14.8)	0.805
5FU and folinic acid	6 (2.8)	5 (3.1)	0.860
Modified de Gramont	1 (0.5)	3 (1.9)	0.192
Capecitabine	19 (8.8)	11 (6.8)	0.475
Bevacizumab	10 (4.6)	12 (7.4)	0.254
Cetuximab	5 (2.3)	5 (3.1)	0.644
Panitumumab	1 (0.5)	6 (3.7)	0.021

The time from diagnosis to surgery was shorter among patients with or without neoadjuvant radiotherapy, but still non-significant statistically. In addition, in 2020 the time for oncologic consultation and to chemotherapy was comparable with the years 2019 without significant changes. Focusing on the treatment type, no significant difference was found regarding rate of surgery, 78 vs. 79%, but significantly higher rate of Xelox protocol were used in 2020 compared to 2019 (21 vs. 13%, *p* = 0.037), in addition higher rate of biological treatment was found in 2020 (14.2 vs. 7.4%, *p* = 0.032).

### Gastric cancer

A total of 60 patients diagnosed with GC were included in the analysis of which 36 (60%) were diagnosed in 2019 and 24 (40%) in 2020. The age adjusted incidence of GC was 5.3 patients per 100,000 residents per year in the year 2019, compared to 5.0 in the year 2020 (*p* = 0.929). No statistically significant differences were found regarding the clinical presenting symptoms, comorbidities, disease stage at diagnosis and mortality rate between patients diagnosed with GC during the COVID-19 year compared with patients diagnosed in the previous year, the results are summarized in Table 2.

**Table 2:** Demographic and clinical characteristics of patients with gastric cancer in the years 2019 and 2020.

**Characteristics**	**Diagnosed 2019**	**Diagnosed 2020**	***P*-value**
	***n* = 36**	***n* = 24**	
Age (mean ±SD)	69.02 ± 13.94	70.0 ± 14.09	0.57
Age at diagnosis (mean ±SD)	67.77 ± 14.28	69.79 ± 14.10	0.47
Gender - male	22 (61)	17 (71)	0.4
Ethnicity Beduoin	5 (14)	1 (4.2)	0.4
**Symptoms**			
Abdominal Pain	19 (54)	11 (46)	0.55
Weight Loss	2 (5.7)	2 (8.3)	0.99
Dysphagia	2 (5.7)	2 (8.3)	0.99
Anemia	7 (20)	7 (29)	0.4
Upper GI Bleeding	5 (14)	2 (8.3)	0.7
**Cancer type**			0.2
Adenocarcinoma	34 (94)	19 (83)	
Signet Ring carcinoma	2 (5.6)	3 (13)	
Linitis Plastica	0	1 (4.3)	
**Localization**			0.8
Antrum	15 (42)	13 (54)	
Body	10 (28)	6 (25)	
Cardia	9 (25)	4 (17)	
Diffuse	2 (5.6)	1 (4.2)	
**Staging**			
I	7 (19.4)	2 (8.3)	0.23
II	12 (33)	8 (33)	>0.9
III	1 (2.8)	3 (12.5)	0.13
IV	9 (25)	11 (37.3)	0.77
X	7 (19)	2 (8.3)	0.29
Death	18 (50)	12 (50)	>0.9
Age at death (mean ±SD)	68.34 ± 22.52	71.01 ± 19.17	0.72
Charlson score (mean ±SD)	6.22 ± 2.52	6.08 ± 3.11	0.82
CIHD	5 (14)	4 (17)	>0.9
CHF	3 (8.6)	2 (8.3)	>0.9
CVA	2 (5.7)	0	0.5
PVD	1 (2.9)	1 (4.2)	>0.9
DM	9 (26)	7 (29)	0.8
COPD	2 (5.7)	1 (4.2)	>0.9
Liver disease	3 (8.6)	1 (4.2)	0.6
CRF	1 (2.9)	0	>0.9
Dementia	1 (2.9)	2 (8.3)	0.6
HTN	21 (60)	13 (54)	0.7
Dyslipidemia	10 (29)	11 (46)	0.2
Smoking	13 (37)	7 (29)	0.5
Obesity	5 (14)	1 (4.2)	0.4

CIHD, Chronic Ischemic Heart Disease; CHF, Congestive heart disease; CVA, cerebrovascular disease; PVD, peripheral vascular disease; DM, diabetes mellitus; COPD, chronic obstructive pulmonary disease; CRF, chronic renal failure; HTN, hypertension.

#### Emergency department visits and hospitalizations

Table 3 presents the emergency department visits, need for hospitalizations, and cause of hospitalization during the 6 months before cancer diagnosis.

**Table 3:** Emergency department visits and hospitalizations among the study groups.

	**Colorectal cancer groups**		
**Characteristics**	**Diagnosed 2019**	**Diagnosed 2020**	***P*-value**
	***n* = 216**	***n* = 162**	
Emergency department visit **During 6 months before diagnosis**	81 (37.5)	71 (43.8)	0.214
Hospitalizations	79 (36.6)	66 (40.7)	0.410
**Causes of hospitalizations**			
Abdominal pain Anemia Bowel obstruction Bowel Perforation Change of bowel habits Lower GI bleeding	25 (32.9) 21 (27.6) 18 (23.7) 4 (3.9) 3 (3.9) 5 (6.6)	18 (25.7) 24 (34.3) 19 (27.1) 2 (2.9) 2 (2.9) 5 (7.1)	0.851
Gastric cancer groups			
**Characteristics**			
Emergency department visits During 6 months before diagnosis	21 (60)	13 (54)	0.656
Hospitalizations	18 (50)	13 (54)	0.752
**Causes of hospitalizations**			0.879
Abdominal pain Anemia Upper GI bleeding Vomiting Weight loss	7 (19.4) 4 (11.1) 4 (11.1) 4 (11.1) 1 (2.8)	4 (16.7) 3 (11.5) 1 (4.2) 4 (16.7) 1 (4.2)	

Higher but not significant emergency department visits and hospitalization were observed in 2020 among CRC patients, 43.8 vs. 37.5%, 40.7 vs. 36.6%, respectively. A lower rate of emergency department visits but a higher rate of hospitalizations in 2020 were found, 54 vs. 60%, 54 vs. 50%, *p* = 0.656 and *p* = 0.752, respectively. In addition, no difference regarding the causes of hospitalizations were found.

### Diagnosis over the years 2019 and 2020

**Figure 2:**
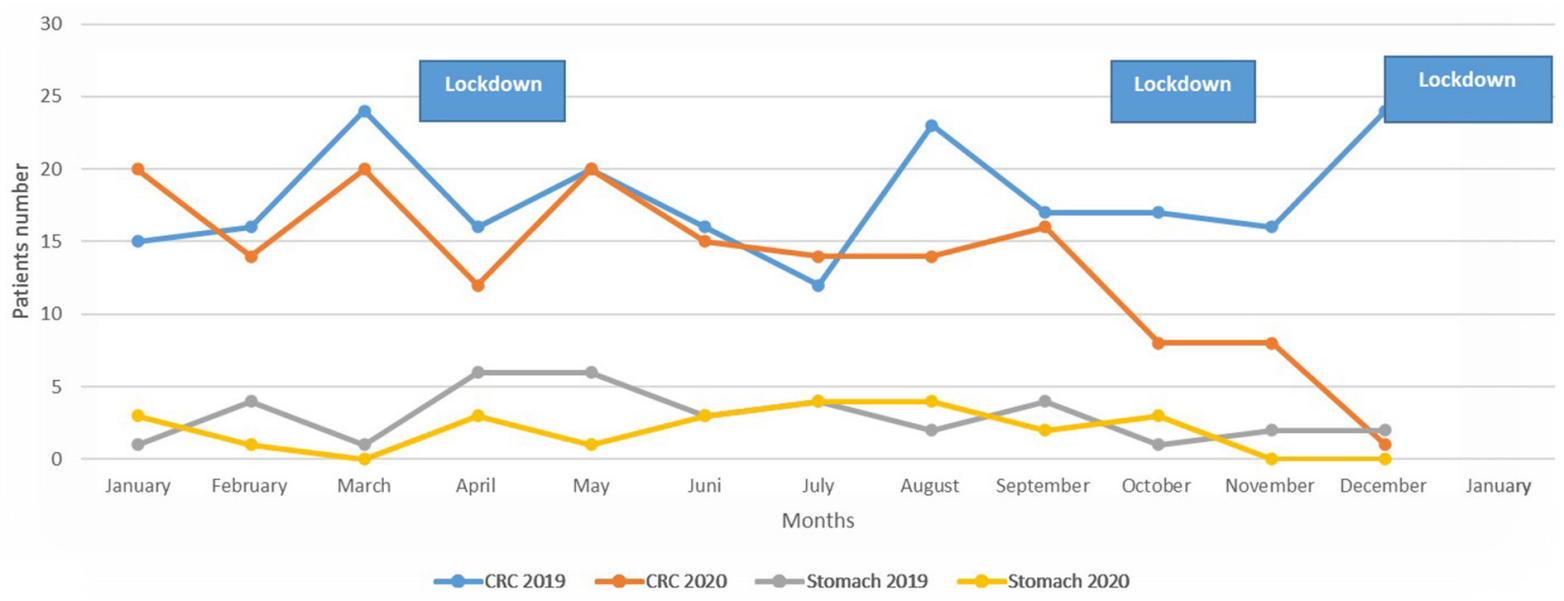
Frequency of diagnosis of CRC and GC according to months.

In Figure 2, the number of patients diagnosed monthly with CRC or GC in the years 2019 and 2020 are presented, charted alongside the periods of national lockdowns in Israel. On a month-by-month comparison no statistically significant differences were observed in the number of patients diagnosed except December 2020 vs. 2019: a clear reduction of case number was observed, the number of patients diagnosed with CRC in 2020 is lower than 2019, decline from 24 cases to one case (*p* < 0.001).

## Discussion

This study demonstrated a significant decrease in the absolute number of patients diagnosed with CRC and GC during the year 2020 (COVID-19 outbreak) compared to the year before (25% for CRC and 33% for GC. In addition, we demonstrated a difference in clinical presentation, but no significant difference noted regarding the disease stage at diagnosis or the all-cause mortality for short term follow up.

The decrease in the age-adjusted incidence for CRC from 29 to 24 per 100,000 residents per years and for GS from 5.3 to 5 per 100,000 residents per year was also not statistically significant. We believe this is a result of the relatively small number of new cases per year per population and although not statistically significant, does represent a true decrease.

Several factors may have contributed to the decrease in incidence of diagnosis of CRC and GC in 2020, including recurrent lockdowns leading to cancellation of endoscopic procedures, particularly at the beginning of the outbreak, fear in the general population for seeking medical services in perceived crowded hospital setting and decreased adherence to screening modalities such as stool testing as screening for CRC and colonoscopy for asymptomatic indications such as family history of CRC, positive fecal occult testing or polyps follow-up.

These results regarding the decrease in diagnosis of CRC or GC are in agreement with other studies that have been published. A study from Italy showed a decrease in detection rates of the high-grade dysplasia/cancers of pancreato-biliary cancer, upper and lower gastrointestinal lesions by 59.1, 70.6, and 68.8% during the lockdown periods in 2020 (6). This study, however, only compared cancer diagnosis rated during their lockdowns vs. regular periods. Notably, another Italian survey-based study collected the number of histologically-proven diagnoses of cancers and showed a decrease of 15.9% in GC, 11.9% in CRC and 9.9% in pancreatic cancer during the COVID crisis (7). Another study from Hong Kong showed a significant decrease in GC and CRC diagnosis, 46.2 and 37% reduction. This study investigated the time period of October 1, 2019 through March 31, 2020 compared to the same period in previous years (8). In our study, the decrease of GC diagnosis was 33% (should be tempered by the low number of cases of GC in our study) and the reduction of CRC diagnosis was 25% in 2020 compared with 2019, which is higher than those reported in the Italian study and more notable given the prolonged period of study. It is also important to mention that our study was more comprehensive and based not only on histological survey, but also on the clinical setting by reviewing the medical records of every patient, yielding important information regarding clinical presentation, disease stage at diagnosis and mortality rates, which makes it of special strength since these are the signs that clinicians will face rather than tissue studies that are taken as confirmation for suspicion of disease.

Regarding the clinical presentations we found that rectal bleeding was of significantly lower frequency as a presenting symptom in the year 2020, compared to the year 2019, while diarrhea was significantly more common and abdominal pain (trending toward significance) in 2020. This change in pattern of clinical presentation of CRC parallels the changes in tumor localization across the colon observed in the study, with rectal bleeding, a common presenting sign of rectal cancer and rectal cancer diagnosis rate, both showing decrease in 2020 compared to 2019. In addition, the shift toward presenting signs that are less alarming (higher frequency of diarrhea and abdominal pain) as opposed to the fewer cases presenting alarming symptoms (rectal bleeding), may have contributed to the overall decline in CRC diagnosis observed during 2020. We propose that the outbreak, particularly in terms of the anxiety of staying at home during lockdowns, resulted in a change of the patients' health services-seeking behaviors.

Focusing on treatment and timing of treatment, in our study no significant differences were found regarding the timing of surgery, oncologist consultation or chemotherapy treatment. In the present study we observed that in 2020 more patients treated with biological treatment than the year before, which could be explained by higher rate of patients with metastatic and advanced disease.

Overall, the effects of such a global crisis lies not only on its impact on procedure's prioritization and health care seeking patterns, but also on understanding of its late effect on cancer staging and mortality. We did not find significant changes in CRC or GC disease stage at diagnosis, eligibility for surgical treatment or mortality rate during the first year of the COVID-19 crisis compared with the previous year. A meta-analytical model the effect of lockdowns on disease staging and mortality as result of screening delay, demonstrated that delays beyond 4–6 months would significantly increase the advanced CRC cases and mortality would increase in cases in which screening was delayed beyond 12 months (9).

The limited time periods of strict lockdowns in our region have led to relatively short interruptions in access to endoscopic screening and diagnostic procedures. This factor may account for the limited impact on disease stage at diagnosis and mortality rates during the first year of the COVID-19 outbreak observed in this study.

Zorzi et al. showed that in a European FIT-based screening program, post-FIT colonoscopy after 9 months was associated with an increased risk of CRC and CRC progression (10). In another national modeling study in the UK, substantial increases in the number of avoidable cancer deaths in England were to be expected as a result of diagnostic delays due to the COVID-19 pandemic (11).

Taken together, our study results confirm findings from other reports on the reduction of CRC and GC during the COVID-19 crisis, but show no significant changes regarding disease stage at diagnosis or mortality rate. These findings should be confirmed by ongoing investigations conducted in the near future. It will be important to investigate the frequency, disease stage at diagnosis and mortality rates of digestive cancer in the year 2021, a year in which the population learned to accommodate with restrictions imposed by the pandemic, and the threshold of fear and anxiety associated with performance of elective medical procedures has probably decreased. In addition, fewer lockdowns and experience-driven change of the government's policies, would likely have decreased the postponement of colonoscopy in 2021 compared to 2020. Findings from our study and several other published models and reports investigating the impact of COVID-19 outbreak on GI cancer detection, may assist professional associations in updating guidelines for endoscopic evaluations during this era for achieving better impact on population health. This may assist physicians to better advise their patients who need endoscopic procedures.

Our study is unique in that it is the first study investigating not only the incidence/number of patients diagnosed with CRC or GS but also comparing the disease stage at diagnosis and mortality.

There are several limitations to our study. First, this is a single-center retrospective study, which accounts for the relatively small number of GC and CRC diagnosed. Inclusion of additional centers would have increased the number of cases and consequently the ability to reach statistical significance in some of the findings in which borderline significance was detected. Second, although SUMC is the only tertiary center in the region, it is possible that some patients opted for treatment at lower lower-level facilities, due to the fear of being subjected to increased risk of infection in large busy hospital. Third, no data regarding treatment abandonment, and treatment modifications were collected. Nonetheless, findings from this study encourage us and hopefully other investigators to expand our efforts for data collection on this topic on ongoing basis, across the country, throughout the globe, such coordinated efforts will contribute to future understanding of how to manage diagnostic endoscopic therapy in pursuit of optimal patient care in times of ongoing and continuously evolving global pandemic.

## Conclusion

A decrease in GC and CRC diagnoses was observed during the year 2020 (COVID-19 crisis) compared with 2019; lower rate of rectal bleeding and higher rate of diarrhea as presenting symptoms were noted in 2020, but no significant difference was found regarding other presenting symptoms, disease stage at diagnosis, eligibility for surgical treatment and or mortality rates.

## Data availability statement

The raw data supporting the conclusions of this article will be made available by the authors, without undue reservation.

## Ethics statement

The studies involving human participants were reviewed and approved by Helsinki Committee - Soroka University Medical Center. Written informed consent for participation was not required for this study in accordance with the national legislation and the institutional requirements.

## Author contributions

NA-F: conceptualization, data curation, methodology, supervision, writing original draft, and project administration. RH and MA-A: data curation and writing–review and editing. TM: methodology, formal analysis, software, and writing–review and editing. BJ and AR: methodology and writing–review and editing. KR: conceptualization, methodology, and writing–review and editing. DS and UN: investigation, methodology, and writing–review and editing. IP: investigation, formal analysis, and writing–review and editing. OE: conceptualization and writing–review and editing. All authors contributed to the article and approved the submitted version.

## Conflict of interest

The authors declare that the research was conducted in the absence of any commercial or financial relationships that could be construed as a potential conflict of interest.

## Publisher's note

All claims expressed in this article are solely those of the authors and do not necessarily represent those of their affiliated organizations, or those of the publisher, the editors and the reviewers. Any product that may be evaluated in this article, or claim that may be made by its manufacturer, is not guaranteed or endorsed by the publisher.
